# Partner involvement in abortion trajectories and subsequent abortion safety in Nigeria and Côte d’Ivoire

**DOI:** 10.1186/s12905-022-02115-z

**Published:** 2022-12-17

**Authors:** Selena Anjur-Dietrich, Elizabeth Omoluabi, Funmilola M. OlaOlorun, Rosine Mosso, Shannon N. Wood, Caroline Moreau, Suzanne O. Bell

**Affiliations:** 1 Department of Population, Family and Reproductive Health, Johns Hopkins Bloomberg School of Public Health, 615 N. Wolfe Street, Baltimore, MD 21205 USA; 2 Centre for Research, Evaluation Resources and Development, Ile-Ife, Nigeria; 3 Department of Community Medicine, University of Ibadan, Ibadan, Oyo Nigeria; 4 École Nationale Supérieure de Statistique et d’Économie Appliquée, Abidjan, Côte d’Ivoire; 5 CESP Centre for Research in Epidemiology and Population Health, INSERM (Institut National de la Santé et de la Recherche Médicale), Villejuif, France

**Keywords:** Unsafe abortion, Abortion trajectories, Partner involvement, Social support

## Abstract

**Background:**

Unsafe abortions contribute to maternal mortality and morbidity worldwide, with disproportionate impacts in lower-income countries. Identifying factors associated with an elevated risk of experiencing an abortion under the most unsafe conditions is an important component of addressing this burden. The partner’s role in obtaining a safe or unsafe abortion is not well understood. This study provides a quantitative assessment of the relationship between partner involvement and subsequent abortion safety.

**Methods:**

The data are drawn from the PMA2020 female surveys and abortion follow-up surveys, fielded in Nigeria and Côte d’Ivoire between 2018 and 2020. The sample includes 1144 women in Nigeria and 347 women in Côte d’Ivoire who reported having ever experienced an abortion. We assess partner involvement in discussing the abortion decision and/or in selecting the method or source and evaluate the relationship between partner involvement and most unsafe abortion (using non-recommended methods from a non-clinical source) versus safe or less safe abortion, adjusting for sociodemographic characteristics.

**Results:**

We find a strong association between experiencing any partner involvement and decreased odds of experiencing a most unsafe abortion (Nigeria: aOR = 0.34, 95% CI 0.26–0.45; Côte d’Ivoire: aOR = 0.27, 95% CI 0.16–0.47). Analyzing the two types of partner involvement separately, we find that partner involvement in the decision is associated with lower odds of most unsafe abortion in both countries (Nigeria: aOR = 0.48, 95% CI 0.39–0.72; Côte d’Ivoire: aOR = 0.34, 95% CI 0.19–0.60); partner involvement in selecting the method and/or source was only significantly associated with lower odds of most unsafe abortion in Nigeria (Nigeria: aOR = 0.53, 95% CI 0.39–0.72; Côte d’Ivoire: aOR = 0.65, 95% CI 0.32–1.32).

**Conclusion:**

In Nigeria and in Côte d’Ivoire, respondents whose partners were involved in their abortion trajectory experienced safer abortions than those whose partners were not involved. These findings suggest the potential importance of including men in education on safe abortion care and persistent need to make safe abortion accessible to all, regardless of partner support.

**Supplementary Information:**

The online version contains supplementary material available at 10.1186/s12905-022-02115-z.

## Background

Out of all abortions that occur globally each year, nearly half (or 25.1 million), are considered unsafe [1]. The WHO defines unsafe abortions as those “carried out by a person lacking the necessary skills or in an environment that does not conform to minimal medical standards, or both” [2]. Unsafe abortions are a major contributor to maternal mortality and morbidity in lower-income countries [1, 3]. Specifically, in sub-Saharan Africa, it is estimated that 10% of maternal mortality is attributable to unsafe abortion, corresponding to approximately 125,000 deaths per year across the region [4]. Understanding the factors informing abortion trajectories is an important component of reducing this burden. One factor that is not yet well understood is how the partner’s involvement contributes to abortion trajectories and safety.

A number of studies have described the ways in which partners, friends, family members, and other individuals can become involved in a person’s abortion experience, with particular emphasis on the role of the partner. Within a relationship, partners’ fertility preferences may or may not concur. These fertility preferences, along with partner dynamics, are likely to shape the decision to continue or terminate the pregnancy, as well as subsequent risks and burdens (including social, health-related, or financial). In many contexts the partner is the most likely person to be involved in discussion about whether to keep the pregnancy [5–7]. Further, stigma associated with premarital sex can preclude disclosure beyond the partner [5]. Less is known, however, about the role of the partner in shaping decision-making beyond the initial discussion regarding the pregnancy outcome.

The body of research describing the role of the partner in abortion experiences is predominantly qualitative and was conducted in several regions (primarily the United States and sub-Saharan African countries), under varied legal conditions, and among largely clinic-based samples [5–20]. This research emphasizes three junctures at which the partner’s influence is particularly salient to a pregnant person: (1) deciding whether to disclose the pregnancy to the partner, (2) deciding, or being told, what to do about the pregnancy, and (3) seeking and obtaining an abortion, including post-abortion care. We use the term “abortion trajectories” to describe the broader abortion experience throughout which partners may become involved. This draws from the conceptual framework proposed by Coast et al. [21], in which abortion trajectories are shaped by individual-level, abortion-specific, and macro-level factors and often progress in a non-linear way (e.g., involving multiple cycles of deliberation, information-gathering, and abortion attempts).

Existing research describes a wide range of experiences among couples (or sexual partners) faced with a pregnancy that is unwanted by one or both parties, from active emotional and logistical support [9, 12–14, 17, 18], to non-involvement [7, 12, 13, 16], to coercive behaviors [8, 9, 12, 19]. Qualitative research from numerous countries suggests that norms around partner involvement in abortion decision-making vary, influencing the first juncture (deciding whether to disclose the pregnancy to one’s partner). Some individuals may also prefer not to disclose the pregnancy to their partner at all to avoid harmful repercussions including intimate partner violence or interference in accessing a wanted abortion, among other concerns [22–25]. The resulting need for secrecy can limit access to safe abortion for reasons related to confidentiality of care-seeking or inability to mobilize necessary resources [7, 12, 19, 21, 23, 26]. Once a person decides to or considers having an abortion (the second juncture), partners are commonly involved in discussing pregnancy options, whether by preference or constrained by social repercussions [5–7]. Discordant partner views on how to manage a pregnancy can impact abortion trajectories in different ways. For example, a person whose partner denies responsibility for the pregnancy may experience this rejection as equivalent to making the decision to have an abortion for them [7, 12, 16, 24]. Partner rejection of a pregnancy may alternatively cause accusations of infidelity from the partner or others made aware of the pregnancy, further motivating abortion [19].

Once the decision has been made to end a pregnancy, pregnant individuals are faced with the challenge of identifying an abortion method and a provider or source for this method, and negotiating access (the third juncture). If the pregnancy was disclosed to the partner, partners may or may not choose to become further involved, and their involvement can take several forms from financial or other logistical support to taking on the role of decision-maker. Among a community-based sample of married men (mostly of low socioeconomic status) in Ibadan, Nigeria, 55% did not believe that they had a role to play in their partner obtaining safe abortion care (SAC) or post-abortion care (PAC) [11]. Among those who did feel they would have a duty towards their partner, the majority believed this to include assisting in obtaining the medication(s) needed. In some cases, partners make decisions about the abortion method and source unilaterally, which may or may not align with the expectations or preferences of the pregnant person and in some cases involves coercion [9, 20]. Partners often provide financial support for the abortion [7, 12, 20], and, less frequently, emotional or spiritual support [5, 8, 10]. Some choose to accompany their partner to the clinic or other provider, though in some settings the heightened social risk of being seen in such a setting makes accompaniment undesirable [12, 20, 27].

Evidence regarding partner involvement in abortion in some cases draws connections to a variety of abortion-related outcomes, including timing of abortion (in a study in Bihar, India) [5] and psychological outcomes such as subjective wellbeing and rating of the abortion experience (in the US) [8]. A community-based sample of married Nepali men whose partners reported having recently had an unintended pregnancy described their involvement as including combinations of: independently making the decision that their partner should terminate the pregnancy; identifying the abortion methods and sources; and accompaniment, payment, and participating in PAC, thereby shaping the abortion experience at the second and third junctures described previously [9]. Respondents in these studies among partners and abortion patients also described a heightened, shared sense of commitment to the relationship as a result of partner accompaniment.

Few studies have so far attempted to link partner involvement to abortion safety, in part due to the limitations of clinic-based recruitment strategies common in abortion research, and to date, no quantitative studies have investigated this potential relationship (see one qualitative study conducted in Zambia [12]). Recruiting large enough samples for quantitative studies focused on sensitive behaviors like abortion can be particularly challenging in contexts where abortion is criminalized and/or highly stigmatized. In this study, we seek to address this gap in the literature by assessing, among people who had an induced abortion (hereafter referred to only as abortion), partner involvement in discussing the decision to have an abortion and in the selection of abortion methods and sources, and to investigate whether this involvement relates to abortion safety in community-based samples of women from Nigeria and Côte d’Ivoire. Both countries criminalize most abortions, and as a result, people seeking abortions in Nigeria and Côte d’Ivoire have a heightened risk of unsafe abortion. Given the limited availability of safe, legal care in these settings, we focus on identifying the role of partner involvement in trajectories leading to abortions that are *most* unsafe: abortions involving non-recommended methods (i.e., methods other than surgery or medication abortion pills) from non-clinical providers [28]. We draw from nationally representative samples of women^1^ of reproductive age in each country to identify individuals who reported ever having had an abortion, allowing us to assess the impact of partner involvement on abortion safety across a wide range of experiences, including abortions that occur entirely outside of formal healthcare systems.

## Methods

### Study context

Nigerian federal law only allows for legal abortion in order to save a woman’s life, and in Côte d’Ivoire, abortion is only legal in order to save a woman’s life or in cases of pregnancy resulting from rape [29]. Despite limited grounds for legal abortion, annual abortion incidences are estimated at 45.8 abortions per 1000 women of reproductive age in Nigeria (95% CI 41.0–50.6) [30] and 40.7 abortions per 1000 women in Côte d’Ivoire (95% CI 33.3–48.1) [31]. Over 60% of abortions in both countries are classified as “most unsafe”, involving non-recommended methods from a non-clinical source [30, 31].

### Data and sampling

This study uses data from PMA2020 in Nigeria and in Côte d’Ivoire. PMA2020 uses a three-stage cluster sampling design in Nigeria (geopolitical zone, state, geographic cluster) and a two-stage cluster sampling design in Côte d’Ivoire (region, geographic cluster), stratified by urban/rural residence to achieve a nationally representative sample of reproductive-aged women in each country (for further sampling information, see: https://www.pmadata.org/data/survey-methodology). We used Nigeria Round 5 and Cote d’Ivoire Round 2 household and female survey data, which were conducted in April–May 2018 and July–August 2018, respectively. These rounds included abortion modules and a subsequent abortion follow-up study.

First, the PMA2020 female survey collected demographic information, and data related to reproductive and family planning history. Embedded within the female survey, the abortion module included questions about community norms related to abortion, and the respondent’s abortion experience. The module used two sets of terminology to ask about abortion history: ever doing something to remove a pregnancy or ever doing something to bring back a late period when respondents were worried that they were pregnant, both of which we considered to be abortions.

All respondents who reported ever having had a completed abortion in the female survey, and who consented to be recontacted, were eligible for the abortion follow-up survey. The follow-up data collection occurred from November 2019–February 2020 in Nigeria and October–November 2020 in Cote d’Ivoire. The survey was not fielded in Kano State in Nigeria due to the scarcity of eligible respondents (44 respondents reported ever having had an abortion). The abortion follow-up surveys in both countries asked respondents additional questions about their only or most recent completed (i.e., successful) abortion. The follow-up survey included questions confirming information about the abortion that was reported in the baseline survey, and about abortion complications, measures of patient-centered quality of care, post-abortion contraceptive use, abortion preferences, how respondents understood the concepts of pregnancy removal vs. period regulation, and awareness of legal status of abortion. It also included questions regarding partner involvement in relation to the first or only abortion method/source sought by the respondent. The full abortion follow-up questionnaires and datasets can be requested via the PMA website (Nigeria: https://datalab.pmadata.org/dataset/doi%3A10349767ty2-va92; Côte d’Ivoire: https://datalab.pmadata.org/dataset/doi:1034976xqy6-nf94).

### Analytic samples

**Fig. 1:**
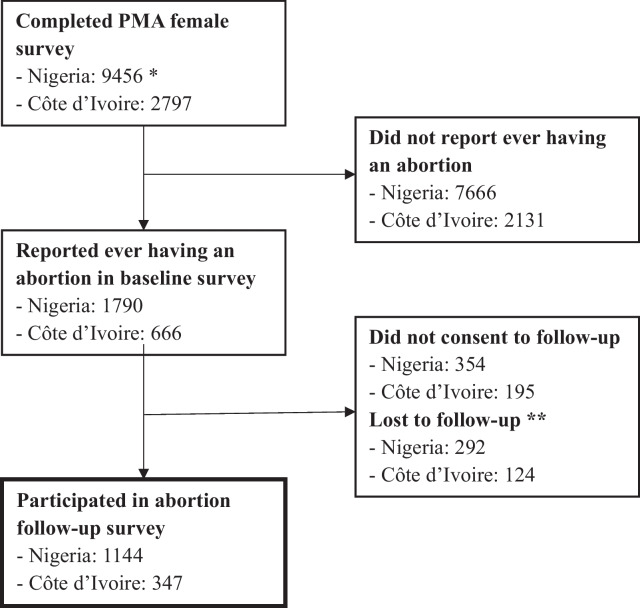
Sample selection flowchart. * This count does not include respondents in Kano State. Due to the scarcity of eligible respondents based on self-report of abortion, Kano State was excluded from recruitment for the abortion follow-up survey. 1759 Kano respondents completed the female survey and 44 reported pregnancy removal or period regulation. ** Nigeria: 14 refused, 2 incomplete, 8 died, 18 did not confirm ever having had an abortion, 88 moved, 45 recruited for a separate study, 117 unknown/other. Côte d’Ivoire: 19 refused, 1 incomplete, 5 died, 16 did not confirm ever having had an abortion, 11 moved, 50 recruited for a separate study, 22 unknown/other

Out of the 1790 respondents who reported ever having had an abortion in the Nigeria Round 5 female survey (excluding respondents in Kano State), 80.2% consented to be recontacted, among whom 79.7% participated in the abortion follow-up survey, resulting in a final sample size of 1144 respondents. In Côte d’Ivoire, 666 women initially reported ever having had an abortion, 70.7% of whom consented to follow-up, and 73.7% of those who consented to be recontacted participated in the abortion follow-up survey for a final sample of 347 respondents (Fig. 1). Out of these final samples, 55 (4.8%) and 14 (4.0%) respondents in Nigeria and Côte d’Ivoire, respectively, were excluded from the regression analyses due to missing data on variables included in the regression models. We use Chi-square tests to assess differences along key study variables between our final analytic sample and those respondents who were eligible but did not complete the abortion follow-up survey.

### Variables

#### Outcome

The primary outcome examined is a “most unsafe” abortion, defined as using a non-WHO-recommended method (any method other than surgical abortion or medication abortion using mifepristone and/or misoprostol) that was provided by a non-clinical source (any source outside of the public or private medical sector) [28]. Non-clinical sources include pharmacies, other shops, friends or relatives, faith-based organizations, traditional healers, or markets/hawkers. Abortions were categorized dichotomously as most unsafe or not, based on method and source used.^2^ Abortions using recommended methods provided by non-clinical sources, using non-recommended methods from clinical sources, and using recommended methods from clinical sources are all coded as abortions that are *not* most unsafe. For women who reported multiple attempts to terminate their pregnancy, we categorized abortion safety based only on the first method/source, as we anticipated the decision surrounding which method/source to use initially would be more likely to be influenced by partner involvement compared to decisions regarding follow-up care for treatment of complications or incomplete abortion.

#### Independent variables

The primary independent variable is partner involvement leading up to the abortion. Respondents were asked about multiple actors’ involvement in making the decision to have an abortion (“Did you talk to any of the following people about the decision to [remove the pregnancy/bring back your period]?”). Respondents were also asked about multiple actors’ involvement in method and/or source selection in two ways: (1) “Before deciding to use [FIRST / ONLY METHOD] from [FIRST/ ONLY PROVIDER], did you seek input or information from any of the following sources?”, and (2) “Who recommended you use this source?” (only for respondents who reported they used a source because it was recommended to them). Multiple select response options for each of these questions included: partner, various family members, friend, health provider, traditional healer, other, or none of the above. For primary analyses, these items were combined into a dichotomous variable, with respondents either being coded as having no partner involvement (if they reported no involvement in any of these items), or having their partner involved (if they reported partner involvement in at least one of these items). In the secondary analysis, we included an indicator for partner involvement in the decision to terminate the pregnancy and a separate indicator for partner involvement in selecting method and/or source (combining the two questions about method and/or source selection) to assess whether specific types of partner involvement play different roles in shaping the abortion trajectory.

#### Covariates

We considered several sociodemographic background characteristics in this analysis that, based on existing literature, plausibly shape both partner involvement and access to safer abortions. These include: age at abortion (categorized as 15–19, 20–29, and 30–49 years)^3^, marital status at abortion (married, not married), residence at abortion (rural, urban), schooling (level attending at time of abortion, or highest attended at interview if not attending at time of abortion; none, primary, secondary, or higher), parity at abortion (no children, any children), wealth tertile at interview (based on results from principal components analysis from information on household assets, building materials, water, and sanitation), and in Nigeria, state at interview (the 6 out of 36 states in which PMA2020 conducted the follow-up interview).

### Statistical analysis

We first conducted univariate and bivariate analyses, and report bivariate distributions of background characteristics and abortion characteristics, by partner involvement (any vs. none) and by abortion safety, per country. Next, we implemented country-specific multiple logistic regression models regressing most unsafe abortion on the binary measure of partner involvement exposure. We subsequently used country-specific unadjusted and adjusted multiple logistic regression models assessing the separate association between most unsafe abortion and the two types of partner involvement (discussing abortion decision and selecting abortion source/method). This model allowed us to explore whether one type of partner involvement may be driving any observed associations between the binary partner involvement indicator and the outcome, accounting for the possibility of partner involvement in both ways.^4^ We also tested for an interaction effect by fitting additional multiple logistic regression models including these same involvement type-specific indicators and including an interaction term for the two types of partner involvement to explore a potential non-additive relationship between experiencing both types of involvement and abortion safety. We further considered the role of gestational age, which may influence both partner involvement and the availability of safe methods (though the causal relationship may work in either direction), by including gestational age categories (< 12 weeks vs. ≥12 weeks) in a multiple logistic regression of most unsafe abortion on binary partner involvement.^5^ Given that the relationship between partner involvement and abortion safety was not qualitatively different from the analyses without gestational age, we present these results in supplemental materials (Additional file 1: Table S1). All analyses were conducted in STATA version 17 [32], with statistical significance set a priori at p < 0.05.

## Results

### Abortion follow-up survey sample

We assessed differences in background characteristics between abortion follow-up survey respondents and respondents who were eligible for the abortion follow-up survey but refused or were lost to follow-up. Respondents who completed the follow-up survey differed significantly from those who did not in the following ways: in both settings, respondents in our sample were significantly more rural, less educated, and less wealthy, and in Nigeria, they differed in geographic distribution (fewer from Lagos, more from Nasarawa and Anambra). We do not have sufficient data to apply the same operationalization of abortion safety to the full sample, as abortion source was only collected for respondents who reported either a surgical abortion or one using any type of pill. But with the available information we have for all respondents, the abortion safety distribution was not statistically significantly different between included and excluded respondents. In Nigeria, included respondents were slightly less likely to have used a recommended method (32% vs. 37%), and in Côte d’Ivoire, we found no difference in recommended method use.

### Descriptive results

Characteristics of respondents who participated in the PMA2020 abortion follow-up surveys in Nigeria and Côte d’Ivoire are summarized in Table 1. Among the 1144 respondents in Nigeria, nearly half (49.3%) were 20–29 years old when they experienced their abortion, and the majority were married (60.6%) and living in urban areas (61.0%) at the time. The majority had attended secondary school or higher (50.9% and 25.9%, respectively), and just over half (55.5%) had children. Among the 347 respondents in Côte d’Ivoire, 44.4% experienced their abortion prior to age 20. At the time of the abortion fewer than half of respondents were married (42.9%), nearly two thirds lived in urban areas (63.4%), about one third had no schooling (31.4%), and 37.8% had only attended primary school. Just over half (57.1%) already had at least one child. Respondents’ household wealth was assessed at the time of interview, and both countries’ samples included more women in the middle and higher wealth tertiles.

**Table 1:** Characteristics of women who reported abortions or menstrual regulation in Nigeria and Côte d’Ivoire

	Nigeria		Côte d'Ivoire	
	N	%	N	%
Background characteristics	(N = 1144)		(N = 347)	
*Age at abortion*				
10–19	249	22.1	152	44.4
20–29	555	49.3	138	40.4
30 +	322	28.6	52	15.2
*Marital status at abortion*				
Married	692	60.6	149	42.9
Not married	449	39.4	198	57.1
*Residence at abortion*^*a*^				
Rural	446	39.0	127	36.6
Urban	698	61.0	220	63.4
*Highest level of school attended*^*b*^				
None	116	10.1	109	31.4
Primary	150	13.1	131	37.8
Secondary	582	50.9	91	26.2
Higher	296	25.9	16	4.6
*Parity at abortion*				
0	509	44.5	149	42.9
1 +	634	55.5	198	57.1
*Wealth tertile at interview*				
Low	341	29.9	99	28.6
Middle	385	33.8	124	35.8
High	414	36.3	123	35.5
*State at interview*				
Anambra	191	16.7	–	–
Kaduna	214	18.7	–	–
Lagos	238	20.8	–	–
Nasarawa	165	14.4	–	–
Rivers	273	23.9	–	–
Taraba	63	5.5	–	–

^a^ Created based on respondent report of having lived in a village (rural) or a town or city (urban) at the time of the abortion

^b^ Based on level of school attending at time of abortion, if attending, or highest level attended according to baseline survey interview. This assumes that respondents who were not attending school at the time of their abortion had already completed their schooling

Table 2 presents characteristics of each respondent’s abortion. In Nigeria, the three most commonly used methods were surgery (27.9%), traditional methods or other methods (27.5%), or pills other than mifepristone and misoprostol (26.6%). In Côte d’Ivoire, the same three methods predominated, but more respondents turned to traditional or other methods (44.1%) than surgical abortion (31.7%); a substantial number of respondents utilized pills other than mifepristone and misoprostol (19.9%). Similar proportions of respondents in Nigeria reported using a private facility, pharmacy, or other source, while only 10% used public facilities, and more than half (53.6%) of respondents in Côte d’Ivoire obtained their abortions from “other”, non-clinical sources. Approximately one in five (21.2% in Nigeria, 18.2% in Côte d’Ivoire) made multiple abortion attempts over the course of their most recent abortion, and just over half experienced a most unsafe abortion (51.6% in Nigeria, 56.5% in Côte d’Ivoire). Partners were more frequently involved in discussing the decision to end the pregnancy compared to method and source selection (Table 2). In Nigeria, 58.9% of partners participated in the discussion while only 39.7% were involved in selecting the method and/or source for the abortion, and in Côte, d’Ivoire, 43.3% participated in the discussion while only 19.0% were involved in selection. The extent of partner involvement in Nigeria was relatively evenly distributed, with 38.4% reporting no involvement, 24.5% reporting one type of involvement, and 37.2% reporting partner involvement in both the decision and method/source selection. Over half (55.0%) of respondents in Côte d’Ivoire reported no partner involvement of any kind, 27.1% of respondents’ partners were involved in only one way (either discussing or selecting), and relatively few (17.9%) were involved in both ways.

**Table 2:** Characteristics of abortion experiences reported by women in Nigeria and Côte d’Ivoire, during first or only attempt

	Nigeria		Côte d'Ivoire	
	N	%	N	%
Abortion characteristics	(N = 1144)		(N = 347)	
*Method*				
Surgery	317	27.9	110	31.7
Mifepristone and/or misoprostol	72	6.3	13	3.7
Other pills	303	26.6	69	19.9
Injection	133	11.7	2	0.6
Traditional methods/other	313	27.5	153	44.1
*Source*				
Public facility	117	10.3	59	17.0
Private facility	366	32.2	85	24.5
Pharmacy/chemist	336	29.6	17	4.9
Other	318	28.0	186	53.6
*Tried multiple things*				
Yes	241	21.2	63	18.2
No	898	78.8	284	81.8
*Abortion was most unsafe*^*a*^				
Yes	585	51.6	196	56.5
No	549	48.4	151	43.5
Years since abortion (mean, SD)	6.4	6.6	9.9	7.0
*Partner involvement in abortion experience*				
No involvement	439	38.4	191	55.0
Involved in one way only:				
Spoke to partner about decision to terminate	248	21.7	88	25.4
Method and/or source selection	32	2.8	6	1.7
Involved in multiple ways	425	37.2	62	17.9

^a^ Abortions categorized as "most unsafe" if using a non-recommended method from a non-clinical provider

### Bivariate analyses

Table 3 presents bivariate associations between partner involvement and sociodemographic and abortion characteristics. In Nigeria, partner involvement was statistically significantly associated with marital status and parity at time of abortion, with the highest proportions of married respondents and those who already had at least one child experiencing any partner involvement. In Côte d’Ivoire, only marital status at abortion was statistically significantly associated with partner involvement, with married respondents having experienced the highest proportion of partner involvement. All abortion characteristics other than whether the respondent made multiple attempts were significantly associated with partner involvement across both contexts. In Nigeria, those who experienced a most unsafe abortion were equally likely to have experienced partner involvement of any kind as to have experienced no partner involvement at all. Among those who experienced an abortion that was not considered most unsafe, partner involvement was much more common compared to non-involvement. In Côte d’Ivoire, those who had a most unsafe abortion were more likely to have had no partner involvement (compared to any partner involvement). We also assessed bivariate associations between most unsafe abortion and sociodemographic and abortion characteristics (Table 4). All background and abortion-related characteristics were statistically significantly associated with abortion safety, apart from marital status in both countries and age and parity at abortion in Nigeria. Most unsafe abortion was more likely among young women and those over age 30 at the time of their abortion, among women living in rural areas, those with less education, and those with less wealth.

**Table 3:** Partner involvement in abortion experience by background and abortion characteristics^a^

	Nigeria (N = 1144)					Côte d'Ivoire (N = 347)				
	No partner involvement		Any partner involvement		p-value	No partner involvement		Any partner involvement		p-value
	N	%	N	%		N	%	N	%	
Total	439	38.2	705	61.8		191	55.0	156	45.0	
*Background characteristics*										
Age at abortion					0.156					0.068
10–19	107	43.0	142	57.0		92	60.5	60	39.5	
20–29	211	38.0	344	62.0		74	53.6	64	46.4	
30 +	113	35.1	209	64.9		22	42.3	30	57.7	
Marital status at abortion					** < 0.001**					** < 0.001**
Married	216	31.2	476	68.8		52	34.9	97	65.1	
Not married	220	49.0	229	51.0		139	70.2	59	29.8	
Residence at abortion^b^					0.817					0.382
Rural	173	38.8	273	61.2		66	52.0	61	48.0	
Urban	266	38.1	432	61.9		125	56.8	95	43.2	
Highest level of school attended^c^					0.688					0.284
None	39	33.6	77	66.4		58	53.2	51	46.8	
Primary	61	40.7	89	59.3		79	60.3	52	39.7	
Secondary	224	38.5	358	61.5		48	52.7	43	47.3	
Higher	115	38.9	181	61.1		6	37.5	10	62.5	
Parity at abortion					**0.003**					0.128
0	219	43.0	290	57.0		89	59.7	60	40.3	
1 +	219	34.5	415	65.5		102	51.5	96	48.5	
Wealth tertile at interview					0.759					0.119
Low	128	37.5	213	62.5		47	47.5	52	52.5	
Middle	153	39.7	232	60.3		76	61.3	48	38.7	
High	155	37.4	259	62.6		67	54.5	56	45.5	
State at interview					0.620					–
Anambra	76	39.8	115	60.2		–	–	–	–	
Kaduna	77	36.0	137	64.0		–	–	–	–	
Lagos	84	35.3	154	64.7		–	–	–	–	
Nasarawa	70	42.4	95	57.6		–	–	–	–	
Rivers	110	40.3	163	59.7		–	–	–	–	
Taraba	22	38.4	41	61.6		–	–	–	–	
*Abortion characteristics*										
Method					** < 0.001**					** < 0.001**
Surgery	74	23.3	243	76.7		42	38.2	68	61.8	
Mifepristone and/or misoprostol	24	33.3	48	66.7		5	38.5	8	61.5	
Other pills	139	45.9	164	54.1		45	65.2	24	34.8	
Injection	41	30.8	92	69.2		0	0.0	2	100.0	
Traditional methods/other	158	50.5	155	49.5		99	64.7	54	35.3	
Source					** < 0.001**					** < 0.001**
Public facility	23	19.7	94	80.3		22	37.3	37	62.7	
Private facility	102	27.9	264	72.1		38	44.7	47	55.3	
Pharmacy/chemist	153	45.5	183	54.5		11	64.7	6	35.3	
Other	157	49.4	161	50.6		120	64.5	66	35.5	
Tried multiple things					0.134					0.711
Yes	82	34.0	159	66.0		36	57.1	27	42.9	
No	353	39.3	545	60.7		155	54.6	129	45.4	
Abortion was most unsafe^d^					** < 0.001**					** < 0.001**
Yes	288	49.2	297	50.8		129	65.8	67	34.2	
No	146	26.6	403	73.4		62	41.1	89	58.9	

Bolded* p*-values indicate significance at* p* < 0.05

^a^ For respondents who made multiple attempts over the course of their abortion experience, only the first attempt is represented in our analyses

^b^ Created based on respondent report of having lived in a village (rural) or a town or city (urban) at the time of the abortion

^c^ Based on level of school attending at time of abortion, if attending, or highest level attended according to baseline survey interview. This assumes that respondents who were not attending school at the time of their abortion had already completed their schooling

^d^ Abortions categorized as "most unsafe" if using a non-recommended method, by a non-clinical provider

**Table 4:** Abortion safety^a^ by background and abortion characteristics (of first/only attempt)

	Nigeria (N = 1144)					Côte d'Ivoire (N = 347)				
	Not most unsafe		Most unsafe		p-value	Not most unsafe		Most unsafe		p-value
	N	%	N	%		N	%	N	%	
Total	549	48.4	585	51.6		151	43.5	196	56.5	
*Background characteristics*										
Age at abortion					0.061					**0.030**
10–19	110	44.5	137	55.5		64	42.1	88	57.9	
20–29	288	52.1	265	47.9		69	50.0	69	50.0	
30 +	144	45.4	173	54.6		15	28.8	37	71.2	
Marital status at abortion					0.528					0.401
Married	336	49.1	348	50.9		61	40.9	88	59.1	
Not married	211	47.2	236	52.8		90	45.5	108	54.5	
Residence at abortion^b^					**0.001**					** < 0.001**
Rural	188	42.4	255	57.6		35	27.6	92	72.4	
Urban	361	52.2	330	47.8		116	52.7	104	47.3	
Highest level of school attended^c^					**0.020**					**0.004**
None	44	38.6	70	61.4		38	34.9	71	65.1	
Primary	75	51.0	72	49.0		52	39.7	79	60.3	
Secondary	270	46.6	309	53.4		50	54.9	41	45.1	
Higher	160	54.4	134	45.6		11	68.8	5	31.2	
Parity at abortion					0.574					**0.026**
0	240	47.5	265	52.5		75	50.3	74	49.7	
1 +	309	49.2	319	50.8		76	38.4	122	61.6	
Wealth tertile at interview					** < 0.001**					** < 0.001**
Low	131	39.0	205	61.0		29	29.3	70	70.7	
Middle	190	49.9	191	50.1		42	33.9	82	66.1	
High	226	54.7	187	45.3		79	64.2	44	35.8	
State at interview					** < 0.001**					–
Anambra	77	40.5	113	59.5		–	–	–	–	
Kaduna	84	40.4	124	59.6		–	–	–	–	
Lagos	143	60.1	95	39.9		–	–	–	–	
Nasarawa	82	50.0	82	50.0		–	–	–	–	
Rivers	136	50.2	135	49.8		–	–	–	–	
Taraba	27	48.4	36	51.6		–	–	–	–	
*Involvement in abortion experience*										
Extent of partner involvement ^d^					** < 0.001**					** < 0.001**
None	146	33.6	288	66.4		62	32.5	129	67.5	
Involved in one way only ^e^	132	47.5	146	52.5		53	56.4	41	43.6	
Involved in decision and method/source selection	271	64.2	151	35.8		36	58.1	26	41.9	
*Partner involved in:*										
Discussing decision	391	58.5	277	41.5	** < 0.001**	85	56.7	65	43.3	** < 0.001**
Selecting method and/or source	281	62.3	170	37.7	** < 0.001**	38	57.6	28	42.4	**0.010**
*Abortion characteristics*										
Tried multiple things					** < 0.001**					** < 0.001**
Yes	469	52.8	420	47.2		137	48.2	147	51.8	
No	79	32.9	161	67.1		14	22.2	49	77.8	

Bolded* p*-values indicate significance at* p* < 0.05

^a^ Most unsafe abortions involve a non-recommended method and a non-clinical provider; all other method and provider combinations are categorized as not most unsafe

^b^ Created based on respondent report of having lived in a village (rural) or a town or city (urban) at the time of the abortion

^c^ Based on level of school attending at time of abortion, if attending, or highest level attended according to baseline survey interview. This assumes that respondents who were not attending school at the time of their abortion had already completed their schooling

^d^ Partners could be involved in any (or none) of the following aspects: decision to terminate, method selection, source selection

^e^ In Nigeria, among respondents whose partners who were only involved in discussing the decision, 127 (51%) experienced a most unsafe abortion while 122 (49%) did not; among those whose partners were only involved in selecting the method/source, most unsafe: 19 (66%) vs. not most unsafe: 10 (34%). In Côte d’Ivoire, among respondents whose partners who were only involved in discussing the decision, most unsafe: 39 (43%) vs, not most unsafe: 51 (57%); among those whose partners were only involved in selecting the method/source, most unsafe: 2 (33%) vs. not most unsafe: 4 (67%)

### Regression analyses

Results from multiple logistic regression models of most unsafe abortion on partner involvement, adjusting for all covariates, are summarized in Table 5. Respondents whose partners were involved in any way (i.e., in discussing the decision and/or selecting the method and/or source) had significantly decreased odds of having a most unsafe abortion in both Nigeria and Côte d’Ivoire (Nigeria: adjusted odds ratio, aOR = 0.34, 95% CI 0.26–0.45; Côte d’Ivoire: aOR = 0.27, 95% CI 0.16–0.47).

**Table 5:** Results from multiple logistic regression of abortion safety (most unsafe vs. other) on respondent characteristics and partner involvement

	Nigeria (N = 1089)^a^				Côte d'Ivoire (N = 333)			
	Adjusted Odds Ratio	95% CI		p-value	Adjusted Odds Ratio	95% CI		p-value
*Partner involvement*								
No partner involvement	*ref*	–	–	–	*ref*	–	–	–
Any partner involvement	**0.34**	**0.26**	**0.45**	** < 0.001**	**0.27**	**0.16**	**0.47**	** < 0.001**
*Age at abortion*								
10–19	*ref*	–	–	–	*ref*	–	–	–
20–29	0.92	0.66	1.29	0.638	0.60	0.33	1.08	0.090
30 +	1.42	0.95	2.13	0.083	1.42	0.59	3.41	0.429
*Marital status at abortion*								
Not married	*ref*	–	–	–	*ref*	–	–	–
Married	1.08	0.72	1.61	0.713	1.13	0.59	2.15	0.718
*Residence at abortion*^*b*^								
Rural	*ref*	–	–	–	*ref*	–	–	–
Urban	0.89	0.65	1.22	0.482	0.59	0.32	1.10	0.095
*Highest level of school attended*^*c*^								
None	*ref*	–	–	–	*ref*	–	–	–
Primary	0.68	0.39	1.18	0.166	0.84	0.46	1.53	0.567
Secondary	0.97	0.58	1.61	0.904	0.84	0.42	1.67	0.620
Higher	0.76	0.43	1.35	0.355	1.13	0.31	4.15	0.858
*Parity at abortion*								
0	*ref*	–	–	–	*ref*	–	–	–
1 +	0.77	0.52	1.14	0.192	1.46	0.78	2.74	0.240
*Wealth tertile at interview*								
Low	*ref*	–	–	–	*ref*	–	–	–
Middle	**0.60**	**0.41**	**0.88**	**0.009**	0.96	0.50	1.83	0.903
High	**0.50**	**0.32**	**0.78**	**0.002**	**0.30**	**0.14**	**0.61**	**0.001**
*State at interview*								
Anambra	*ref*	–	–	–	–	–	–	–
Kaduna	0.80	0.49	1.30	0.370	–	–	–	–
Lagos	**0.48**	**0.32**	**0.73**	**0.001**	–	–	–	–
Nasarawa	**0.44**	**0.26**	**0.73**	**0.001**	–	–	–	–
Rivers	**0.58**	**0.39**	**0.88**	**0.009**	–	–	–	–
Taraba	0.63	0.33	1.21	0.167	–	–	–	–

Bolded* p*-values indicate significance at* p* < 0.05

Both models demonstrated goodness of fit (Nigeria: Hosmer-Lemeshow chi-square with 8df = 3.94, p = 0.863; Côte d’Ivoire: Hosmer-Lemeshow chi-square with 8df = 2.50, p = 0.962)

^a^ Ns do not match Tables 1-4 due to missingness

^b^ Created based on respondent report of having lived in a village (rural) or a town or city (urban) at the time of the abortion

^c^ Based on level of school attending at time of abortion, if attending, or highest level attended according to baseline survey interview. This assumes that respondents who were not attending school at the time of their abortion had already completed their schooling

Assessing the two types of partner involvement (involvement in the decision to end the pregnancy, involvement in selecting method and/or source), we tested for associations with each type of partner involvement separately, and in an additional model further included an interaction term for respondents whose partners were involved in both ways. The interaction term was not statistically significantly associated with most unsafe abortion. We report the results of the regression with no interaction term in Table 6, providing unconditional main effects of each type of involvement, and accounting for the other type of involvement. In Nigeria, each type of partner involvement was independently, significantly associated with reduced odds of most unsafe abortion (involvement in decision: aOR = 0.48, 95% CI 0.35–0.66; involvement in method and/or source selection: aOR = 0.53, 95% CI 0.39–0.72). In Côte d’Ivoire, partner involvement in the decision to end a pregnancy was significantly associated with much lower odds of most unsafe abortion (aOR = 0.34, 95% CI 0.19–0.60), while involvement limited to selecting abortion method and/or source was not statistically significantly associated with the outcome (aOR 0.65, 95% CI 0.32–1.32). In both countries, respondents whose partners were involved in both ways had a very low (and statistically significant) odds ratio of most unsafe abortion (Nigeria: aOR = 0.25, 95% CI 0.19–0.34; Côte d’Ivoire: aOR = 0.22, 95% CI 0.11–0.46).

**Table 6:** Results from multiple logistic regression of abortion safety (most unsafe vs. other) on respondent characteristics and two domains of partner involvement

	Nigeria (N = 1089)^a^				Côte d'Ivoire (N = 333)			
	Adjusted Odds Ratio	95% CI		p-value	Adjusted Odds Ratio	95% CI		p-value
*Partner involved in decision to terminate*								
Yes	*ref*	–	–	–	*ref*	–	–	–
No	**0.48**	**0.35**	**0.66**	** < 0.001**	**0.34**	**0.19**	**0.60**	** < 0.001**
*Partner involved in selecting method and/or source*								
Yes	*ref*	–	–	–	*ref*	–	–	–
No	**0.53**	**0.39**	**0.72**	** < 0.001**	0.65	0.32	1.32	0.234
*Age at abortion*								
10–19	*ref*	–	–	–	*ref*	–	–	–
20–29	0.93	0.67	1.30	0.692	0.60	0.33	1.09	0.093
30 +	1.41	0.94	2.11	0.099	1.50	0.63	3.60	0.364
*Marital status at abortion*								
Not married	*ref*	–	–	–	*ref*	–	–	–
Married	1.15	0.77	1.72	0.498	1.12	0.59	2.14	0.733
*Residence at abortion*^*b*^								
Rural	*ref*	–	–	–	*ref*	–	–	–
Urban	0.87	0.63	1.20	0.392	0.59	0.32	1.09	0.092
*Highest level of school attended*^*c*^								
None	*ref*	–	–	–	*ref*	–	–	–
Primary	0.70	0.40	1.21	0.202	0.85	0.47	1.55	0.604
Secondary	1.01	0.60	1.69	0.964	0.81	0.40	1.61	0.546
Higher	0.81	0.45	1.44	0.472	1.12	0.30	4.12	0.869
*Parity at abortion*								
0	*ref*	–	–	–	*ref*	–	–	–
1 +	0.75	0.51	1.12	0.162	1.41	0.75	2.64	0.286
*Wealth tertile at interview*								
Low	*ref*	–	–	–	*ref*	–	–	–
Middle	**0.62**	**0.42**	**0.91**	**0.014**	0.96	0.51	1.83	0.907
High	**0.51**	**0.32**	**0.79**	**0.003**	**0.30**	**0.14**	**0.61**	**0.001**
*State at interview*								
Anambra	*ref*	–	–	–	–	–	–	–
Kaduna	0.81	0.50	1.32	0.420	–	–	–	–
Lagos	**0.48**	**0.32**	**0.74**	**0.001**	–	–	–	–
Nasarawa	**0.48**	**0.29**	**0.81**	**0.005**	–	–	–	–
Rivers	**0.59**	**0.39**	**0.88**	**0.011**	–	–	–	–
Taraba	0.67	0.35	1.30	0.239	–	–	–	–

Bolded* p*-values indicate significance at* p* < 0.05

Both models demonstrated goodness of fit (Nigeria: Hosmer–Lemeshow chi-square with 8df = 4.36, p = 0.823; Côte d’Ivoire: Hosmer–Lemeshow chi-square with 8df = 2.50, p = 0.962)

^a^Ns do not match Tables 1, 2, 3 and 4 due to missingness

^b^Created based on respondent report of having lived in a village (rural) or a town or city (urban) at the time of the abortion

^c^Based on level of school attending at time of abortion, if attending, or highest level attended according to baseline survey interview. This assumes that respondents who were not attending school at the time of their abortion had already completed their schooling

## Discussion

Respondents whose partners were involved in their abortion trajectory, either by discussing the decision to end the pregnancy or in method/source selection, had significantly lower odds of experiencing a most unsafe abortion in both Nigeria and Côte d’Ivoire. Respondents in Nigeria whose partners were involved in discussing the decision to end the pregnancy or selecting the method and/or source used had 0.34 times the odds of experiencing a most unsafe abortion compared to those whose partners were not involved, adjusting for background characteristics (*p* < 0.001). In Côte d’Ivoire, the reduction in odds of most unsafe abortion among respondents whose partners were involved in any way was even more evident (aOR = 0.27, *p* < 0.001). Partner involvement has been linked to psychological benefits for both parties, but its relationship to abortion safety has previously not been quantitatively assessed; these results contribute to the literature on partner involvement in abortion by documenting a strong association between partner involvement and improved abortion safety in two West African contexts. Our results also show that some degree of partner involvement is a common, but not universal experience in both study countries, with 61.8% of respondents in Nigeria and 45.0% in Côte d’Ivoire experiencing any partner involvement.

In both countries, partners were much more commonly involved in discussing the decision to terminate the pregnancy as compared to involvement in selecting the method and/or source for the abortion. Though we are unable to directly determine whether the partner accepted responsibility for the pregnancy, these results may reflect rejection of the pregnancy by the partners, and the common experience of seeking abortions alone as a result, as documented in existing research [7, 12, 16]. Alternatively, partners may simply lack information about potential methods or sources to contribute. In Nigeria it was more common for a partner to be involved in both ways than in just one way, and in Côte d’Ivoire a small minority (17.9%) of respondents’ partners were involved in both ways.

Several factors may contribute to the different patterns of involvement reported in Nigeria and in Côte d’Ivoire and should be investigated in future studies. A greater proportion of respondents in Nigeria were married at the time they had an abortion and experienced some or multiple forms of partner involvement, and in Côte d’Ivoire, the majority of unmarried respondents experienced no partner involvement at all. This may reflect a preference for non-disclosure of the pregnancy among unmarried people who want an abortion, often due to the corresponding expectation for partner support, or lack thereof [7, 19, 20, 24]. The distribution of ages at the time of the abortion also differs notably, with double the proportion of respondents in Côte d’Ivoire reporting on abortion that occurred before age 20 (44.4% vs. 22.1% in Nigeria). Adolescents face heightened barriers to abortion access and distinct norms governing what is considered acceptable sexuality and childbearing. Younger partners and those in less established partnerships (either in duration or quality) may not feel equipped or obligated to assist with these challenges [11].

In addition to investigating the relationship between *any* partner involvement and abortion safety, we also modeled the role of the two different types of involvement (in discussing abortion, and in method/source selection) independently. This analysis explored whether one particular type of involvement may be driving the association between any partner involvement and abortion safety. In both countries, partner involvement in the decision to have an abortion is significantly associated with improved abortion safety. Studies on the role of the partner in abortion trajectories highlight the extraordinary difficulty of accessing safe abortion care in secret, specifically without alerting the partner involved in the pregnancy [7, 12, 21]. Respondents who do not discuss their decision to have an abortion with their partner may be in a position where they need to keep the abortion, or even the pregnancy, a secret from their partner, and may be faced with more limited options for abortion care [26].

The lack of significant, independent association in Côte d’Ivoire between abortion safety and method/source selection may be due to the rarity of respondents reporting that partners were involved in method/source selection only (n = 6), though the adjusted odds ratio is much lower for involvement in the discussion than in method/source selection. In Nigeria, these measures are both significant and of similar magnitudes. Together, these results suggest that both types of involvement included in this analysis can contribute to the relationship between partner involvement and abortion safety, though their distribution and relative importance may vary by context. Prior studies in Nigeria and Côte d’Ivoire find that adolescents, poor women, and women with no education are disproportionately more likely to experience most unsafe abortions [30, 31]. In addition to the secrecy constraints noted above, partner involvement may introduce additional knowledge of safer abortion options and financial or material resources to facilitate access.

This study is not without limitations. Population-based abortion research is limited by ubiquitous under-reporting of abortions, the extent of which has been assessed in the United States [33] but not yet in Nigeria or Côte d’Ivoire. Out of the representative survey samples from which our abortion follow-up sample is drawn, 16% of respondents in Nigeria and 24% in Côte d’Ivoire reported ever having had an abortion; however, we do not have external estimates of lifetime abortion prevalence for comparison. People who self-report their abortions in a survey may be systematically different in terms of partner involvement and abortion safety than those who do not disclose and would therefore be ineligible for the abortion follow-up survey. It is possible, for example, that women who did not disclose either their pregnancy or abortion to (and therefore involve) their partner are less willing to disclose their abortion in a survey, and/or are less likely to make the disclosures that may be required on the path to obtaining safer care, which would bias our results. There was also a large amount of attrition among the full group of respondents who reported ever having had an abortion in the baseline surveys (38% lost in Nigeria, 48% lost in Côte d’Ivoire), and we noted several systematic differences between respondents who were able and willing to be re-contacted several years after the baseline survey (however, these observed differences are in part accounted for in our multivariate analyses).

Our analysis focuses on the beginning of the trajectory, but eligibility is limited to those who eventually completed their abortion. We are unable to examine those individuals who attempted an abortion and did not try again if it was unsuccessful, and who would have been exposed to what we classify here as most unsafe conditions. People whose abortions were so unsafe that they led to their death are not represented in this sample. Across sub-Saharan Africa, there are 520 deaths per 100,000 unsafe abortions [4]; however, this group is small compared to the larger population of people who have abortions and unlikely to change the observed associations.

The survey instrument also introduced several limitations. Based on the questions asked in the follow-up survey, we cannot conclude that improved abortion safety is a result of *wanted* partner involvement, while the literature on partner involvement describes both supportive and coercive experiences. We also expect that partner involvement in abortion trajectories exists along a spectrum (e.g., discussing a decision at length and addressing questions and concerns vs. a brief agreement on the pregnancy outcome), but in this study, we only measure associations with the presence or absence of two types of involvement. Further, we cannot determine whether unsafe options were chosen as a result of denial of financial support due to limited survey items related to financing the abortion. Our measure of marital status includes those “living with a man as if married”, which corresponds to measures used in related literatures but may not ideally represent the importance of marital status in shaping partner involvement in abortion trajectories. Additionally, wealth is measured at the household level at the time of interview and may therefore be shaped in part by hardships endured as a result of experiencing a most unsafe abortion.

The data may include abortions reported with more or less certainty or accuracy due to decreasing recall over the years, and our analysis groups together abortions occurring in different contexts as abortion access (particularly using mifepristone and misoprostol) and norms regarding partner involvement in health have changed over the past several decades. However, when we tested the same relationships restricting to abortions occurring within 5 years of the survey (57% in Nigeria and 36% in Côte d’Ivoire), findings were consistent with our analysis that included all abortions. Self-reported data also creates the potential for misclassification of abortion method, specifically for misreporting medication abortion pills as unknown pill types; however, due to interviewer training on procedures for identifying common types of pills available in each setting, we do not expect our results to be significantly impacted by such misclassification. Further, in Nigeria only 1% of respondents reported using an unknown pill, while in Côte d’Ivoire, 17% did.

This study also has several strengths. Our analysis drew from a nationally representative sample of women, as opposed to using the facility-based sampling approach that most existing partner involvement studies employ. This allowed us to include those who do not seek safe abortion care or post-abortion care from a clinical source. This study also used a large, quantitative sample as opposed to smaller qualitative studies, which make up the majority of the literature on this topic in low-resource settings. Finally, we used data from two West African contexts that are similar in terms of abortion incidence, legality, and safety; the relative consistency in our findings indicates that the impact of partner involvement may be comparable across settings with similar abortion restrictions, stigma, and provider contexts.

These findings and their limitations have several implications for future research. Further studies should investigate the range of experiences between wanted and unwanted partner involvement, include a broader range of types of involvement (particularly financial support), address how gestational age at pregnancy discovery shapes partner involvement, and survey men as well as women about participation in abortion trajectories, acknowledging that men have a stake and particular power in deciding pregnancy outcomes. Future research should also investigate the role of partner involvement in other abortion-related outcomes such as quality of care and whether people are able to access their preferred method for an abortion and assess these relationships in different types of legal and social settings.

## Conclusion

Leveraging a large sample of women who have had abortions, from a variety of clinical and nonclinical providers, we find that partner involvement is strongly associated with lower odds of most unsafe abortion. This may indicate either that partners afford pregnant people a greater ability to navigate limited options for care (potentially due to greater financial, educational, or social resources), or that people seeking abortions without their partner’s involvement have a more constrained set of options, potentially due to a need for secrecy, the stigma associated with pregnancy outside of a union, or both. Future research should explore these possible mechanisms for the relationship between partner involvement and abortion safety. Our study cannot distinguish between wanted support and coercion or unwanted involvement, and existing research on abortion trajectories emphasizes a broad range of influences shaping abortion care-seeking. However, for those people whose partner’s involvement was welcomed, our findings suggest that men are an important population to include in education on family planning, including abortion. Abortion education in legally restrictive settings may be challenging; however, laws in Nigeria and in Côte d’Ivoire permit abortion in some situations, justifying further sharing of information about safe abortion. Crucially, our findings reflect a persistent need to make safe abortion care accessible to all, independent of a partner’s support.

## Supplementary Information

**Additional file 1: Table S1.** Results from multiple logistic regression of abortion safety (most unsafe vs. other) on gestational age, respondent characteristics and partner involvement. **Table S2.** Abortion method and source during first or only attempt reported by women in Nigeria (N=1144); shaded cells represent "most unsafe" abortions

## Data Availability

Data for this study will be made publicly available at pmadata.org (expected by January 2022), at which point anyone can download the dataset for the female questionnaire and abortion follow-up surveys after completing a brief request form (https://www.pmadata.org/data/available-datasets).

## Footnotes

1 While people other than women (i.e. trans men and gender non-conforming or gender-expansive individuals) also have abortions and are at risk of most unsafe abortions, PMA2020 female survey recruitment is limited to individuals who identified as women in the household survey.

2 PMA2020 identifies commonly available types of pills and various names associated with each during country-specific survey piloting. Interviewers are trained to record the appropriate pill category based on the specific type or brand name reported by the respondent, and may show photos of common pills to assist the respondent in identifying the correct pill. The categories of pills include: misoprostol and/or mifepristone, fever-reducing pills (e.g. antibiotics and anti-malarials), emergency contraception, contraceptive pills, other pills that do not fit these categories, or unknown if the respondent cannot remember what they took. Any reported surgical procedure (manual vacuum aspiration, dilation and curettage, dilation and evacuation) was recorded by interviewers as a surgical abortion. Other methods involving traditional procedures (e.g. uterine massage, herbs, inserting foreign objects into the vagina) were recorded as traditional methods, not surgical abortion.

3 Age categories were selected to allow for sufficient numbers of respondents in each category across datasets, and correspond broadly to different stages in the life course of respondents (adolescence, primary childbearing years, and beyond).

4 We use the *lincom* command in Stata to calculate odds ratios and associated confidence intervals comparing respondents whose partners were involved in both ways to those whose partners were not involved at all.

5 The mean gestational age at abortion was 7.1 weeks (SD = 5.5 weeks) in Nigeria and 8.7 weeks (SD = 5.5 weeks) in Côte d’Ivoire.
