# Supplementary material for: Partner involvement in abortion trajectories and subsequent abortion safety in Nigeria and Côte d’Ivoire
Source: BMC Womens Health. 2022 Dec 17;22:530. doi: 10.1186/s12905-022-02115-z (PMC9759876; doi:10.1186/s12905-022-02115-z)
Supplement: Supplementary file 1 — Additional file 1: Table S1. Results from multiple logistic regression of abortion safety (most unsafe vs. other) on gestational age, respondent characteristics and partner involvement. Table S2. Abortion method and source during first or only attempt reported by women in Nigeria (N=1144); shaded cells represent "most unsafe" abortions [file 12905_2022_2115_MOESM1_ESM.docx]

**Supplementary material:** Partner involvement in abortion trajectories and subsequent abortion safety in Nigeria and Côte d’Ivoire

| **Supplemental Table 1. Results from multiple logistic regression of abortion safety (most unsafe vs. other) on gestational age, respondent characteristics and partner involvement** | | | | | | | | |
| --- | --- | --- | --- | --- | --- | --- | --- | --- |
|  |  |  |  |  |  |  |  |  |
|  | **Nigeria (N=1089)*** | | | | **Côte d'Ivoire (N=333)*** | | | |
|  | **Adjusted Odds Ratio** | **95% CI** | | **p-value** | **Adjusted Odds Ratio** | **95% CI** | | **p-value** |
| **Partner involvement** |  |  |  |  |  |  |  |  |
| No partner involvement | *ref* | -- | -- | -- | *ref* | -- | -- | -- |
| Any partner involvement | **0.36** | **0.28** | **0.47** | **<0.001** | **0.27** | **0.16** | **0.47** | **<0.001** |
| **Gestational age** |  |  |  |  |  |  |  |  |
| ≤12 weeks | *ref* | -- | -- | -- | *ref* | -- | -- | -- |
| >12 weeks | **0.56** | **0.39** | **0.80** | **0.001** | 1.10 | 0.63 | 1.94 | 0.730 |
| **Age at abortion** |  |  |  |  |  |  |  |  |
| 10-19 | *ref* | -- | -- | -- | *ref* | -- | -- | -- |
| 20-29 | 0.91 | 0.65 | 1.27 | 0.567 | 0.61 | 0.34 | 1.11 | 0.108 |
| 30+ | 1.43 | 0.95 | 2.16 | 0.084 | 1.46 | 0.60 | 3.50 | 0.403 |
| **Marital status at abortion** |  |  |  |  |  |  |  |  |
| Not married | *ref* | -- | -- | -- | *ref* | -- | -- | -- |
| Married | 1.09 | 0.72 | 1.64 | 0.690 | 1.02 | 0.53 | 1.96 | 0.954 |
| **Residence at abortion**** |  |  |  |  |  |  |  |  |
| Rural | *ref* | -- | -- | -- | *ref* | -- | -- | -- |
| Urban | 0.89 | 0.65 | 1.23 | 0.488 | 0.59 | 0.31 | 1.12 | 0.105 |
| **Highest level of school attended***** |  |  |  |  |  |  |  |  |
| None | *ref* | -- | -- | -- | *ref* | -- | -- | -- |
| Primary | 0.62 | 0.35 | 1.09 | 0.096 | 0.85 | 0.47 | 1.56 | 0.607 |
| Secondary | 1.00 | 0.59 | 1.68 | 0.989 | 0.89 | 0.44 | 1.79 | 0.740 |
| Higher | 0.75 | 0.42 | 1.34 | 0.333 | 1.16 | 0.31 | 4.30 | 0.822 |
| **Parity at abortion** |  |  |  |  |  |  |  |  |
| 0 | *ref* | -- | -- | -- | *ref* | -- | -- | -- |
| 1+ | 0.77 | 0.51 | 1.15 | 0.197 | 1.53 | 0.81 | 2.91 | 0.194 |
| **Wealth tertile at interview** |  |  |  |  |  |  |  |  |
| Low | *ref* | -- | -- | -- | *ref* | -- | -- | -- |
| Middle | **0.62** | **0.42** | **0.91** | 0.014 | 0.94 | 0.49 | 1.79 | 0.842 |
| High | **0.49** | **0.31** | **0.77** | **0.002** | **0.29** | **0.14** | **0.61** | 0.001 |
| **State at interview** |  |  |  |  |  |  |  |  |
| Anambra | *ref* | -- | -- | -- | -- | -- | -- | -- |
| Kaduna | 0.86 | 0.53 | 1.41 | 0.559 | -- | -- | -- | -- |
| Lagos | **0.49** | **0.32** | **0.75** | **0.001** | -- | -- | -- | -- |
| Nasarawa | **0.50** | **0.30** | **0.84** | **0.009** | -- | -- | -- | -- |
| Rivers | **0.60** | **0.39** | **0.90** | **0.014** | -- | -- | -- | -- |
| Taraba | 0.61 | 0.32 | 1.18 | 0.141 | -- | -- | -- | -- |
| Both models demonstrated goodness of fit (Nigeria: Hosmer-Lemeshow chi-square with 8df =10.41, p=0.237; Côte d’Ivoire: Hosmer-Lemeshow chi-square with 8df =1.91, p=0.984). | | | | | | | | |
| *Ns do not match Tables 1-4 due to missingness | | | | | | | | |
| **Created based on respondent report of having lived in a village (rural) or a town or city (urban) at the time of the abortion | | | | | | | | |
| ***Based on level of school attending at time of abortion, if attending, or highest level attended according to baseline survey interview. This assumes that respondents who were not attending school at the time of their abortion had already completed their schooling. | | | | | | | | |

| **Supplemental Table 2. Abortion method and source during first or only attempt reported by women in Nigeria (N=1144); shaded cells represent "most unsafe" abortions** | | | | |
| --- | --- | --- | --- | --- |
|  | **Source** | | | |
|  | Public facility | Private facility | Pharmacy or chemist | Other |
| **Method** |  |  |  |  |
| Surgery | 46 (4.0*) | 256 (22.6) | 9 (0.8) | 6 (0.5) |
| Mifepristone and/or misoprostol | 11 (1.0) | 9 (0.8) | 51 (4.5) | 1 (0.1) |
| Other pills | 32 (2.8) | 34 (3.0) | 224 (19.8) | 13 (1.2) |
| Injection | 24 (2.1) | 62 (5.5) | 41 (3.6) | 5 (0.4) |
| Traditional methods/other | 4 (0.4) | 4 (0.4) | 11 (1.0) | 291 (25.7) |
| * Percentage of total sample represented by each method and source combination | | | | |

| **Supplemental Table 3. Abortion method and source during first or only attempt reported by women in Côte d'Ivoire (N=347); shaded cells represent "most unsafe" abortions** | | | | |
| --- | --- | --- | --- | --- |
|  | **Source** | | | |
|  | Public facility | Private facility | Pharmacy or chemist | Other |
| **Method** |  |  |  |  |
| Surgery | 45 (13.0) | 65 (18.7 | 0 (0) | 0 (0) |
| Mifepristone and/or misoprostol | 5 (1.4) | 1 (0.3) | 5 (1.4) | 2 (0.6) |
| Other pills | 7 (2.0) | 16 (4.6) | 12 (3.5) | 34 (9.8) |
| Injection | 0 (0) | 2 (0.6) | 0 (0) | 0 (0.0) |
| Traditional methods/other | 2 (0.6) | 1 (0.3) | 0 (0) | 150 (43.2) |
| * Percentage of total sample represented by each method and source combination | | | | |
